# Notch signaling through Tramtrack bypasses the mitosis promoting activity of the JNK pathway in the mitotic-to-endocycle transition of Drosophila follicle cells

**DOI:** 10.1186/1471-213X-6-16

**Published:** 2006-03-16

**Authors:** Katherine C Jordan, Valerie Schaeffer, Karin A Fischer, Elizabeth E Gray, Hannele Ruohola-Baker

**Affiliations:** 1 Department of Biochemistry, Box 357350, 1959 NE Pacific Street, University of Washington, Seattle, WA 98195, USA

## Abstract

**Background:**

The follicle cells of the *Drosophila *egg chamber provide an excellent model in which to study modulation of the cell cycle. During mid-oogenesis, the follicle cells undergo a variation of the cell cycle, endocycle, in which the cells replicate their DNA, but do not go through mitosis. Previously, we showed that Notch signaling is required for the mitotic-to-endocycle transition, through downregulating String/Cdc25, and Dacapo/p21 and upregulating Fizzy-related/Cdh1.

**Results:**

In this paper, we show that Notch signaling is modulated by Shaggy and temporally induced by the ligand Delta, at the mitotic-to-endocycle transition. In addition, a downstream target of Notch, *tramtrack*, acts at the mitotic-to-endocycle transition. We also demonstrate that the JNK pathway is required to promote mitosis prior to the transition, independent of the cell cycle components acted on by the Notch pathway.

**Conclusion:**

This work reveals new insights into the regulation of Notch-dependent mitotic-to-endocycle switch.

## Background

The cell cycle in developing organisms is intricately orchestrated by extrinsic signals [1-3]. Different signaling pathways probably define the rate-limiting cell cycle steps in different cell types, thus providing and explanation for why different cancers target different tissues. Thus, understanding the control of cell cycle is critical for understanding both development and carcinogenesis.

Studies on a natural variant of the mitotic cycle, the endocycle, have revealed how signaling pathways negatively regulate cell cycle [4,5]. The endocycle, as seen in megacaryocytes, trophoblasts, and *Drosophila *nurse and follicle cells, among other tissues, is a variation of the normal cell cycle in which rounds of DNA replication and growth occur without intervening mitoses [6]. The key question in endocycle regulation is how the transition from the mitotic phase to the endocycle is controlled. Two signaling pathways have been identified as regulators of the mitotic-to-endocycle transition: the thrombopoetin pathway, which acts during differentiation of megakaryocytes, and the Notch pathway, which acts during *Drosophila *oogenesis and during the differentiation of trophoblasts [7-11,6]. Human teratocarcinomas also arise from defects in the mitotic-to-endocycle transition in trophoblasts [12]. The cell cycle targets of these pathways have remained elusive until very recently [13,14].

The Notch pathway is used for cell-cell communication throughout development. The basic components of the pathway are the Notch receptor, the two Notch ligands, Delta and Serrate, the transcription factor Suppressor of Hairless (Su(H)), and the bHLH transcription factors encoded by the *Enhancer of Split *complex genes, *E*(*spl*) [15-18]. In *Drosophila *follicle cells, the Notch pathway functions in the mitotic-to-endocycle transition and differentiation [8,9]. Specifically, the ligand Delta is secreted by germ line cells and activates Notch in the follicle cells. The cytoplasmic portion of Notch is subsequently cleaved and moves to the nucleus where, in combination with Su(H), it affects the transcription of various target genes.

Lack of Notch activity in *Drosophila *follicle cells leads to prolonged mitosis at the expense of endocycles. This result suggests that Notch functions in this context as a tumor suppressor [8,9]. Interestingly, recent work on the mouse Notch1 protein has also revealed a tumor suppressor function for the Notch pathway [19-22].

In the follicle epithelium, Notch regulates three cell cycle genes: a G2/M regulator Cdc25 phosphatase, String (STG); a regulator of the APC ubiquitination complex Cdh1/Fizzy-related (FZR); and an inhibitor of the CyclinE/CDK complex, Dacapo (DAP) [8,13,14]. Notch activity leads to downregulation of String and Dacapo, and upregulation of FZR. Here we describe components that determine how Notch controls these cell cycle targets: sgg modulates Notch protein, Delta expression regulates the timing of Notch activation, and the transcription factor Tramtrack controls Notch-dependent cell cycle regulation. We also show that the JNK-pathway induces mitotic- and represses endo-cycles, independent of the cell cycle regulators acted on by Notch pathway.

## Results

**Figure 1:**
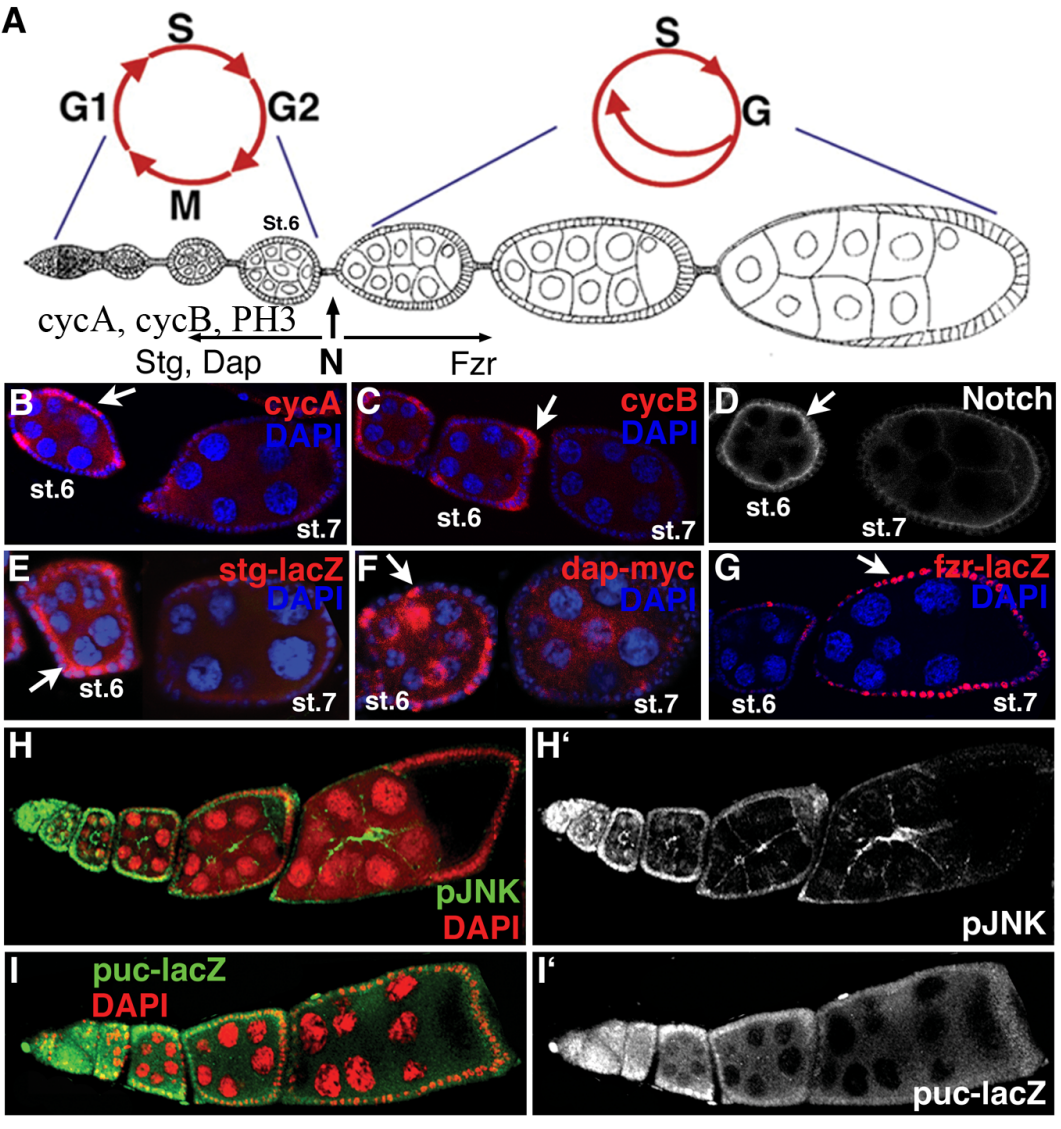
Transition in epithelial follicle cells from mitotic cycle to endocycle during Drosophila oogenesis. (A) Signaling from the Notch pathway causes follicle cells surrounding each oocyte to cease mitosis and begin endocycling at stage 6–7. (B) Cyclin A, (C) Cyclin B and (A) PH3 expression through stage 6 indicates the mitotically active follicle cells. (D) Notch protein is also expressed on the apical side of the follicle cells through stage 6 (arrow) and is cleared from the plasma-membrane upon activation. (E) String (stg), as shown by stg-lacZ (6.4 kb) promoter construct and (F) dacapo (dap), as shown by dap-5gm promoter construct, are both expressed in follicle cells prior to the transition and down-regulated in response to Notch signaling. (G) Fizzy-related (fzr), as shown by Fzr-lacZ enhancer trap line *fzrG0326*, is expressed in follicle cells after the transition. (H and H') Phosphorylated JNK staining (green) is upregulated in the follicle cells prior to the mitotic-to-endocycle transition in WT egg chambers. All cells are labeled by DAPI (red). (I and I') Puc-LacZ (green), a reporter construct for Puckered (*puc*^*A251*^), expression is upregulated in follicle cells during mitosis. All cells are marked with DAPI (red).

During Drosophila oogenesis, each 16-cell group of germline cells is encapsulated by follicle cells and separated from the next successive egg chamber in the germarium. The follicle cells continue to divide through stage 6 of oogenesis, at which point they cease mitosis and begin to endocycle [23](Fig. 1A). The abrupt end of mitosis after stage 6 is evident in the lack of mitotic markers, CycA, CycB, and PH3, at later stages (Fig. 1A,B,C). Notch activation in follicle cells by its ligand Delta from the germ line results in the cessation of mitosis and the promotion of endocycle [8,9]. The proper activation of Notch also results in processing and clearance of the Notch receptor from the apical membrane of follicle cells past stage 6 [9,8](Fig. 1D). The Notch signaling pathway regulates cell cycle components; Notch down-regulates *string *and *dacapo *and upregulates *fzr *[9,24,8](Fig. 1E–G). In addition, Notch down-regulates the cell adhesion molecule FAS3 [8,13,14](Fig. 5). To determine how Notch regulates the cell cycle targets, we screened for new components of the process and found genes of two categories: those that regulate Notch pathway activity (*shaggy*, *disheveled *and *tramtrack*) and those of the JNK-pathway.

### Components of the wingless pathway, shaggy and disheveled but not pygopus control cell cycle activity

**Figure 2:**
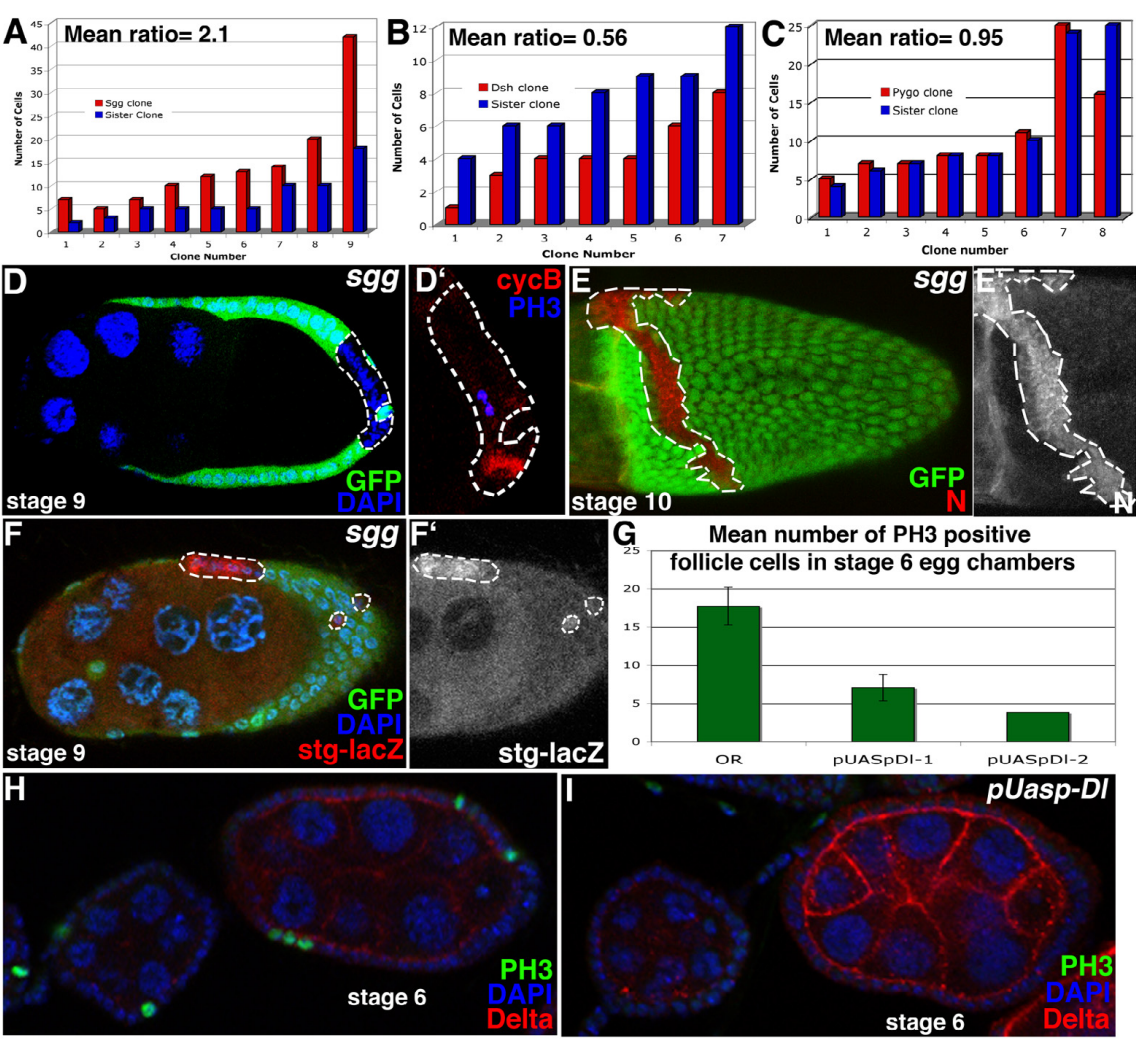
Shaggy (*sgg*^*M1-1*^) and Disheveled (*dsh*^*477*^), components of the wingless pathway, are involved in cell cycle control, but Pygopus (*pygo*) is not. (A) Quantification of the number of cells in Sgg mutant clones compared to the number in their sister clones shows that they are twice as large. (B) Quantification of the number of cells in Dsh mutant clones compared to the number in their sister clones shows that they are half the size of their twin spots (p = 0.0005). (C) However, quantification of the number of cells in Pygo mutant clones compared to the number in their sister clones shows that there is no affect on cell cycle when Pygo expression is lost. (D, D') Sgg clones (dashed line) show prolonged expression of CycB (red) and PH3 (blue) indicating that mitoses are still occurring in this stage 9 egg chamber. After the mitotic-to-endocycle transition, Sgg mutant clones show upregulation of Notch (E, E') and stg-lac Z (F, F'). (G) Premature expression of pUASpDelta-1 and pUASpDelta-2 causes a 60% and 89% reduction in the number of PH3 positive cells respectively (n = 37 *matTub-Gal4; pUASpDl-1*; n = 10 *pUASpDl-2/matTub-Gal4*; n = 30 control). (H) In control ovarioles, PH3 (green) is expressed at stage 6. (I) Driving Delta (red) prematurely in the germline causes all or some of the follicle cells to cease division early (as visualized by an absence of PH3 positive cells). Clones are marked with loss of GFP and indicated by dashed lines.

Our candidate gene approach revealed that *shaggy *(*sgg*), a GSK3-kinase and *disheveled *(*dsh*) are required for the mitotic-to-endocycle transition. Follicle cell clones for *shaggy *continue mitotic division after the mitotic-to-endocycle transition and are twice the size of their respective twin spots (mutant/wt = 2.1, n = 9, p = 10^-2^; Fig. 2A, D). These cells continue to express mitotic markers, cycB and PH3 past stage 6 when wild type cells abruptly cease the expression of these markers (Fig. 2D'). In contrast, *dsh *clones are on average half the size of their respective twin spots (mutant/wt = 0.56, n = 7, p = 5 × 10^-4^; Fig. 2B), suggesting that dsh is required for mitotic division.

Shaggy and Dsh can act in two different capacities; in some developmental contexts these proteins act in Wingless signaling, while in others they directly control the Notch pathway. In the *Drosophila *wing, the Notch and wingless pathways control cell proliferation synergistically through downregulation of *string *[25,26]. Dsh can also antagonize the Notch pathway by binding the Notch C-terminal domain [27], and Shaggy, a GSK-3 kinase, affects Notch signaling through phosphorylation of the intracellular domain of Notch [28,29]. To dissect in which capacity Shaggy and Dishevelled act in follicle cell division control, we first tested whether the canonical wingless-pathway was acting in the process by analyzing the follicle cells mutant for a transcription factor in the canonical wingless pathway, Pygopus [30,31]. Mutant clones for *pygopus *do not affect cell cycle (Fig. 2C; mutant and wild type clones are the same size and the cells are polyploid), suggesting that the canonical wingless pathway is not required for mitotic-to-endocycle transition. These results suggest that in this context, Sgg and Dsh act independently of the canonical wingless pathway.

### Notch protein is regulated by sgg, while temporal activation of the pathway is controlled by Delta

Given that sgg and dsh affect mitosis, but do not act in the canonical *wingless *pathway in this context, we tested whether *shaggy *mutant clones directly affect Notch protein. Upon activation at stage 6 in wild-type follicle cells, Notch protein level is highly reduced by stage 7 [9,24,8](Fig. 1D). In *sgg *clones, however, Notch protein levels are not reduced at the apical side of follicle cells during the mitotic-to-endocycle transition, and remain high later in oogenesis (Fig. 2E, E'). This result is reminiscent of the maintenance of Notch protein observed when the activation by Delta is lacking [9], suggesting that Notch protein is not activated and therefore not degraded in *sgg *mutant clones. Furthermore, cells mutant for *sgg *fail to downregulate the Notch responsive 6.4 kb *string *promoter reporter construct, *string-lacZ *after stage 6 of oogenesis (Fig. 2F), a phenotype similar to the one observed in *Notch *mutant clones. In addition, defects in Fas3 down-regulation are observed in *sgg *clones. These results suggest that modification of Notch by Shaggy is required for Notch activation and thus the correct transition of follicle cells from mitosis to endocycle as well as proper down-regulation of Fas3.

Since both Shaggy and Delta are required for Notch activity at the mitotic-to-endocycle transition, we tested whether one of these components is sufficient to activate Notch, and thus the transition to endocycle, if expressed prematurely. In wild type ovarioles, Delta expression is observed at the time of the transition, suggesting that Delta expression might regulate the timing of Notch activation. To test this hypothesis, we expressed Delta in the germline during early oogenesis (*pUASpDl-2/matTub-Gal4 *or *matTub-Gal4/+; pUASpDl-1/+*). Premature expression of Delta resulted in a dramatic reduction in the number of mitotically dividing follicle cells. The number of stage 6 egg chambers with normal, mitotically dividing follicle cells, marked by PH3 staining was reduced to half (Fig. 2G,H, I). Therefore, premature expression of Delta in the germline is sufficient to activate the Notch pathway and cease mitotic divisions in the follicle cells. Nevertheless, this does not rule out the possibility that another factor in addition to the timing of Delta expression is required for full activation of the Notch pathway at the mitotic-to-endocycle transition.

### Tramtrack mimics Notch activity in cell cycle regulation

**Figure 3:**
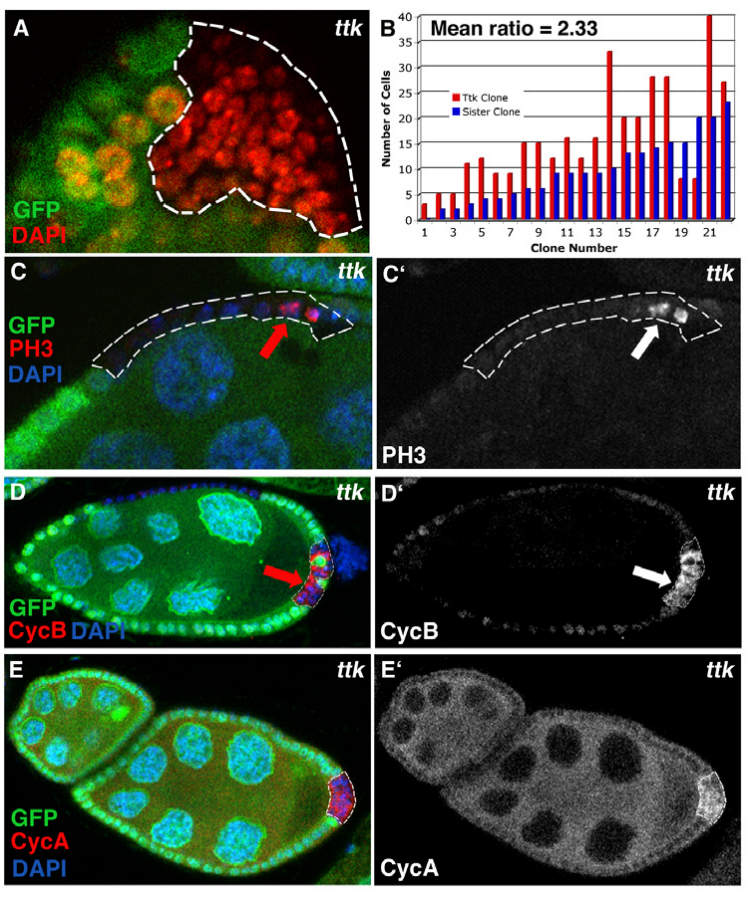
Tramtrack (ttk) is required in follicle cells for the mitotic-to-endocycle transition. (A) *Ttk*^*1e11 *^mutant clones marked by loss of GFP (indicated by dashed line) show a decrease in the size of cell nuclei, marked with DAPI (red), indicating a defect in the mitotic-to-endocycle transition of those cells. (B) Quantification of the number of cells in *ttk *clones compared to the number in their sister clones shows that on average they are twice as large, which implies that *ttk *mutant cells have undergone an extra round of mitotic division (mean = 2.33, p = 0.0006). (C) *ttk *mutant clones show prolonged expression of mitotic markers, PH3 (C, C'), Cyc B (D, D') and Cyc A (E, E'; 25% n = 32, 60% n = 30, 50% n = 22, respectively).

A candidate gene approach also revealed that the Zn-finger transcription factor Tramtrack(Ttk) is required for the proper mitotic-to-endocycle transition. As in *Notch *and *Su*(*H*) clones, prolonged mitotic division is observed in *ttk *clones; instead of large endocycling nuclei, small mitotic nuclei were detected in *ttk *clones (68%, n = 34; Fig. 3A, dashed area). These *ttk *mutant cells continue to express mitotic markers, PH3, CycB, and CycA after the transition; signs of extra cell division resulting in twice the amount of cells observed in the wild type clones (mutant/wt = 2.33, p = 6 × 10^-4^; Fig. 3B–E).

**Figure 4:**
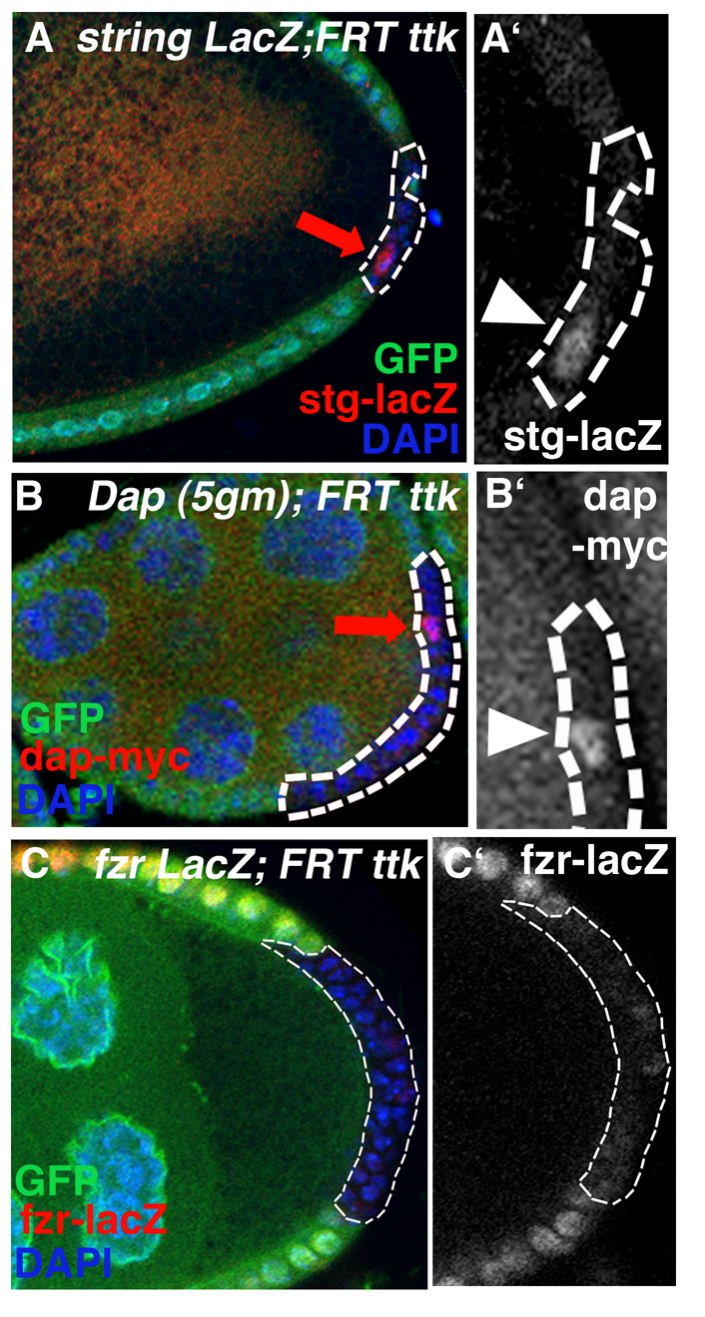
Tramtrack affects targets of the Notch pathway involved in the mitotic-to-endocycle transition. *Ttk *mutant clones past stage 6–7 mitotic-to-endocycle transition show upregulation of *stg-lacZ *(A, A'; arrow, red, s.10 egg chamber, *stg 6.4 kb *promoter construct, 37% n = 27) and *dap-myc *(B, B'; arrow, red, s.8 egg chamber, *dacapo5gm *promoter construct, 64% n = 25) and down-regulation of *Fzr-lacZ *(C, C'; red, s.9 egg chamber, *fzrG0326*, 70% n = 123). All cells are marked with DAPI (blue), GFP is green.

Ttk is a target of Notch activation in sensory organ precursor development [32-34]. To test whether Ttk also acts with Notch in the mitotic-to-endocycle transition, we analyzed whether Notch cell cycle targets, String, Dacapo and FZR (Fig. 1E–G) were affected in *ttk *clones. The regulation of all three Notch cell cycle targets was defective in *ttk *clones; expression of *stg-lacZ *and *dap-myc *was prolonged after the transition and *fzr-lacZ *expression was reduced, indicating that the cells do not progress to endocycle at the transition (Fig. 4A–C; 37% n = 27, 64% n = 25 and 70% n = 23, respectively).

**Figure 5:**
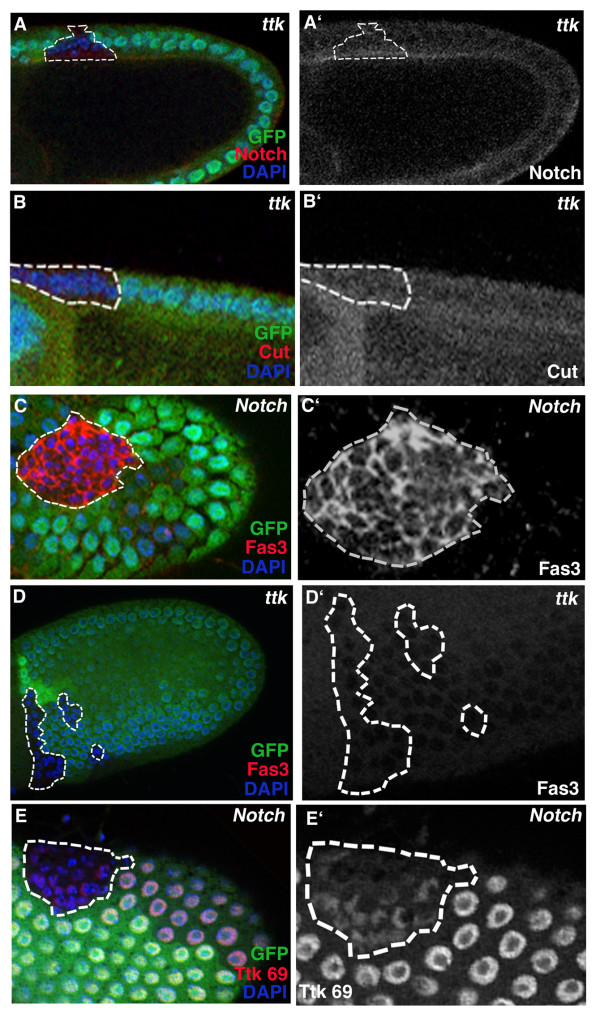
Tramtrack, downstream of Notch, is involved in cell cycle regulation but not in cell differentiation. (A, A') Notch is processed and cleared normally from the apical side of follicle cells in *ttk*-clones. (B, B') Cut down-regulation is normal in *ttk*-clones (23/23, except in the most posterior region, Supp Fig. 1; Althauser et al., 2005). *N *mutant clones (C, C') but not *ttk *mutant clones (D, D') show ectopic expression of FAS3 after the transition, indicating that Ttk does not act in Notch dependent follicle cell differentiation that involves Fas3. (E, E') *N *mutant clones (stage 9 egg chamber) show reduction of *ttk *expression (red), indicating that Ttk is downstream of N. However, this result does not rule out the possibility that Ttk acts in a parallel pathway. All cells are marked with DAPI (blue), GFP is green, clones are marked with dashed lines.

### Tramtrack is not required for Notch dependent, Fas3 marked cell differentiation

To test which stage of the Notch pathway Ttk is affecting, we first analyzed whether the processing of Notch protein was abnormal in the *ttk *clones, as was the case in follicle cell *sgg *clones and germline *Delta *clones [9](Fig. 1D). Apical clearing of Notch after activation is normal in *ttk *clones (Fig. 5A), suggesting that Notch protein is normally activated and processed. In addition, we tested the expression of Cut protein whose Notch dependent down-regulation is critical for mitotic-to-endocycle transition [35,36]. Cut-protein is normally down-regulated in *ttk*-clones (except in the polar cell precursor group), suggesting that Ttk acts independently or downstream of Cut (Fig 5B, n = 23, Additional File 1)[37].

Defects in Notch and Cut activity also result in ectopic expression of Fasciclin 3 (Fas3), a homophilic adhesion molecule, indicating that the Notch pathway at least partially affects differentiation as well as cell cycle [8,9,36](Fig. 5C). In contrast, *ttk *clones (outside the most posterior region [37]) do not show ectopic Fas3 expression (Fig. 5D; 51/52) suggesting that *tramtrack *at this developmental stage acts on cell division, independent of Fas3 marked cell differentiation.

*ttk *clones show division defects and abnormal regulation of the Notch cell cycle targets but do not affect Notch activation or down-regulation of Cut (Fig. 3, 4, 5B). To test whether Ttk is a downstream component of Notch activation, we analyzed Ttk protein in Notch clones and observed a reduction of Ttk69 protein levels in most of the clones (Fig. 5E). These results are consistent with the hypothesis that Ttk acts downstream of Notch in the mitotic-to-endocycle transition. However, at this point we can not rule out the possibility that Ttk acts through a parallel pathway.

These data suggest that in addition to acting downstream of Notch in sensory organ precursor division, Ttk functions downstream of Notch in cell cycle control, independent of Notch-controlled cell differentiation in the general follicle cell layer.

### JNK pathway controls follicle cell division prior to the mitotic/endocycle transition

From the expression screens we found that an enhancer trap line for the *puckered *gene, *puckered-LacZ*, shows expression in mitotically active follicle cells [13](Fig. 1I). Puckered is a target of the Jun kinase (JNK) pathway, and encodes a JNK phosphatase involved in a negative feedback loop in the pathway [38]. In addition, antibodies to the phosphorylated form of JNK show expression in mitotic follicle cells (Fig. 1H), indicating that the JNK pathway might control mitotic cycling.

**Figure 6:**
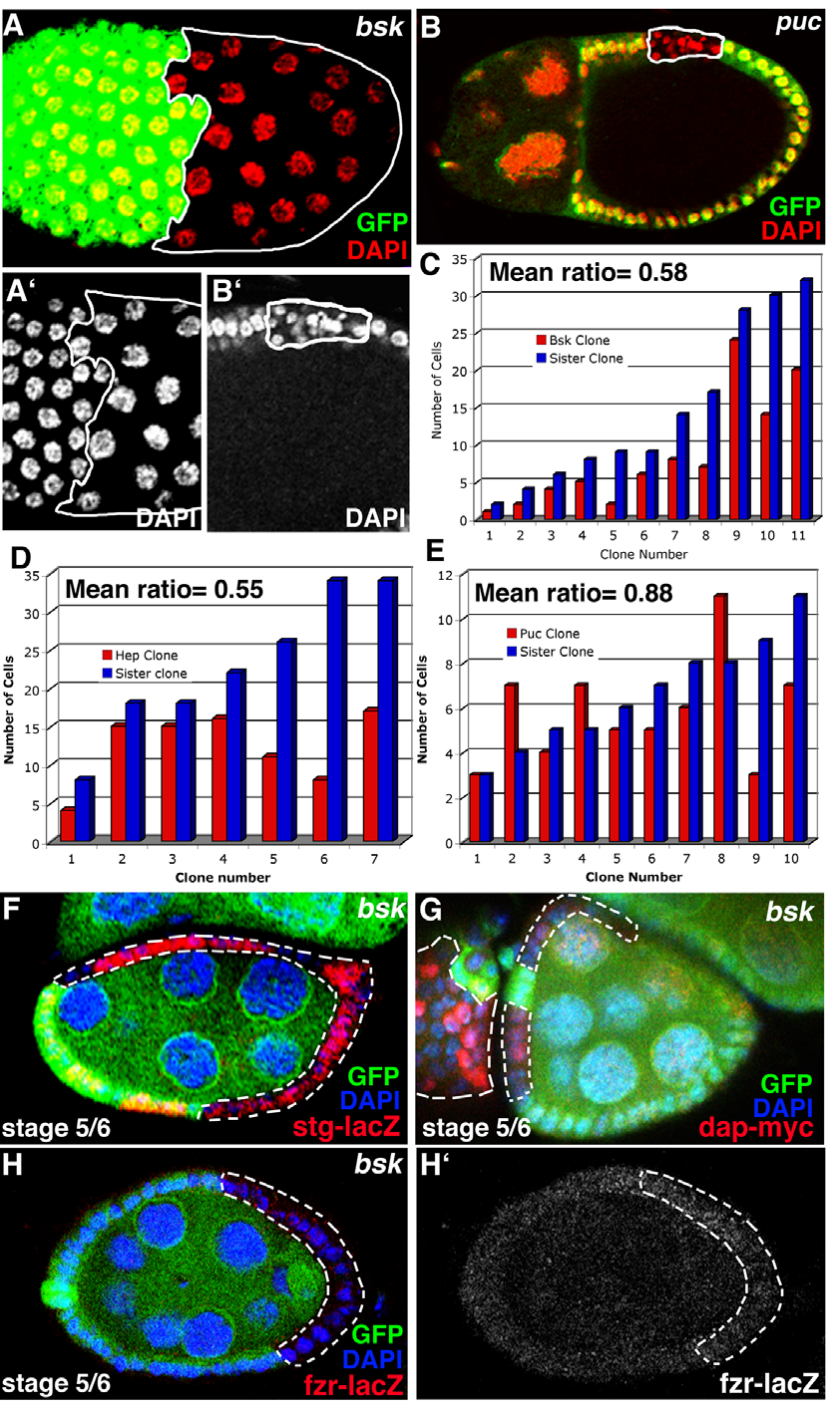
JNK pathway components Basket (*Bsk*^*170B*^), Puckered (*Puc*^*A251*^), and Hemipterous (*Hep*^*r75*^) control cell cycle in follicle cells by promoting mitosis prior to the transition to endocycle. (A, A') Bsk mutant cells (no green GFP) have larger nuclei, marked with DAPI (red), compared to wild-type cells marked with GFP (green). (B, B') Puc mutant cells (no green GFP) appear to be disrupted in mitotic division but the cell nuclei, marked with DAPI (red), are not abnormally large and therefore have not gone into endocycle. (C) Quantification of the number of cells in Bsk mutant clones compared to the number in their sister clones shows that the clones are approximately half the size of their sister clones, indicating a premature exit from mitotic division (mean = 0.58, p = 0.002). (D) Quantification of the number of cells in Hep mutant clones compared to the number in their sister clones shows a similar phenotype (mean = 0.55, p = 0.02). (E) Quantification of the number of cells in Puc mutant clones compared to the number in their sister clones shows no consistent defect in mitotic division (mean = 0,88, p = 0.4, suggesting that the size difference between sister and mutant clones is not statistically significant). (F-H) Bsk mutant cells (no green GFP) compared to wild-type cells marked with GFP (green) in stages prior to the mitotic-to-endocycle show no affects on (F) string expression, marked with LacZ (red), or (G) dacapo expression, marked with c-Myc tag (red). (H, H') Bsk mutant cells (no green GFP) also do not show premature fizzy-related expression, marked with LacZ (red). All cells are marked with DAPI (blue).

To test whether the JNK pathway is involved in the mitotic-to-endocycle transition, we made follicle cell clones for *basket *(Drosophila JNK), *puckered*, and *hemipterous*, the kinase that phosphorylates JNK. *basket *clones cease mitotic division too early, resulting in clones that contain approximately half the number of cells as their respective twin spots (mutant/wt = 0.58, n = 11, p = 2 × 10^-3^; Fig. 6A,C). In addition, the cells in *basket *clones have larger nuclei than the neighboring wild type cells (Fig. 6A'). This phenotype has been observed before and has been interpreted as extra endocycles [39,14,36]. These data suggest that cells mutant for *basket *stop mitotic cycling and enter endocycles prematurely. *hemipterous *clones show a similar phenotype, also ceasing mitotic division too early (mutant/wt = 0.55, n = 7, p = 2 × 10^-2^; Fig. 6D), although they do not enter endocycle. Clones for the negative component of the pathway, *puckered*, show no consistent defect in mitotic cycles between stages 1–6 in oogenesis. However the nuclei in *puckered *mutant cells remain small, suggesting that these follicle cells do not enter endocycles at the mitotic-to-endocycle transition (Fig. 6B, B', E). Instead, some of the puckered mutant cells might be blocked in the transition state and/or undergo apoptosis while a few continue mitotic cycling [40]. These data indicate that the JNK pathway in *Drosophila *is required for the promotion of mitosis in follicle cells.

### Targets of JNK pathway activity are different from Notch pathway targets

As discussed, Fizzy-related, String and Dacapo are targets of the Notch pathway in the regulation of the mitotic to endocycle transition; Notch pathway activity is required to downregulate the expression of String and Dacapo, and upregulate the expression of FZR at the transition from mitosis to endocycle [8,13,14](Fig. 1E–G). We made clones for *basket *to test if the JNK pathway regulates the same genes as the Notch pathway in cell cycle control. Since *basket *clones enter endocycle prematurely, we expected premature down-regulation of String and Dacapo, and up-regulation of FZR. However, clones for basket do not affect the expression of *string-lacZ, dap-myc *or *fzr-lacZ *(Fig. 6F–H, respectively). Thus, although the JNK pathway is required for mitosis, it does not regulate the same targets as the Notch pathway.

## Discussion and Conclusion

Notch controls the mitotic-to-endocycle transition in follicle epithelial cells; Notch pathway activity arrests mitotic cell cycle and promotes endocycles by downregulating *string*/cdc25 and *dacapo*/p21, and upregulating *fzr*/Cdh1. Here, we identify components regulating this transition, Delta, Shaggy, and Tramtrack. Shaggy and Delta are required for the activation of Notch protein. However, Delta is sufficient to activate Notch in this process, since premature expression of Delta in the germline stops mitotic division of the follicle cells. We have now identified the transcription factor Tramtrack as a connection between Notch and the cell cycle regulators *stg, fzr*, and *dap*. Loss of Tramtrack function phenocopies the Notch and Su(H) phenotypes; overproliferation and misregulation of cell cycle components. However, high FAS3 expression, indicative of differentiation defects in Notch clones, is not observed in *ttk *clones, suggesting that Tramtrack might regulate a branch of the Notch pathway specific for cell cycle control. We also show that the JNK-pathway is a critical mitosis promoting pathway in follicle cells. Loss of JNK(*bsk*) or JNKK(*hep*) activities stop follicle cell mitotic cycles, while loss of JNK promotes premature endocycles. In addition, loss of the negative regulator of the pathway, the phosphatase Puckered, results in a lack of endocycles. However, the Notch-responsive cell cycle targets that, in combination, can induce the mitotic-to-endocycle transition, *stg*, *fzr*, and *dap*, are not regulated by the JNK-pathway.

### Shaggy regulates Notch processing while Delta regulates the timing of Notch activity

Notch signaling is highly regulated throughout development [41]. The Notch receptor can be regulated by glycosylation of the extracellular domain, as well as by endocytosis and degradation of the intracellular domain, thus affecting the activity of the pathway. Shaggy has been shown to phosphorylate and thus affect the stability of Notch protein [28,29]. Here we show that normal processing and clearing of Notch protein from the apical surface of follicle cells upon Notch activation does not occur in *shaggy *clones, indicating that Notch is not normally activated and therefore regulation of the downstream targets does not take place.

In many organisms and tissues the Notch ligands are ubiquitously expressed and thus not likely to regulate Notch pathway activation. However, at the mitotic to endocycle transition, Delta is upregulated in the germline, making ligand expression a likely candidate for regulation of Notch activity. Here we show that premature expression of Delta in the germline can cause mitotic division to stop at least one stage earlier than in control ovarioles. Nonetheless, this effect is seen in only half of the ovarioles. Therefore, it is possible that yet another process is regulating Notch activity at the transition in addition to Delta expression. Further testing will determine if endocytosis of Notch might also regulate Notch activity at the mitotic-to-endocycle transition. One possible protein is Numb, which regulates Notch in human mammary carcinomas, indicating that Numb may have a more general role in cell cycle control than just the division of the sensory organ precursors [42].

### Tramtrack regulates mitotic-to-endocycle transition

The fact that Notch overrides the mitotic activity of the JNK pathway by acting on cell cycle regulators that can induce the mitotic-to-endocycle transition puts further demand on understanding the connection between Su(H) and cell cycle regulators. We have identified one such component, the transcription factor Tramtrack. Two Tramtrack proteins exist, Ttk69 and Ttk88, both of which are affected by the allele we used in these studies [43]. However, staining with antibodies specific to the two forms reveals that only Ttk69 is detectable in the follicle cells and downregulated in Notch clones.

Ttk69 can control proliferation in glial cells [44], strengthening its candidacy for a critical component between Notch and cell cycle controllers in follicle epithelial cells. In addition, the Ttk-like BTB/POZ-domain zinc-finger transcription repressor in humans is Bcl-6, a protein associated with B-cell lymphomas [45].

We have now analyzed Ttk function in the follicle cell mitotic-to-endocycle transition and have shown that the Notch-responsive cell cycle components *stg*, *dap*, and *fzr *are responsive to Ttk function. Interestingly, Ttk69 controls the *string *promoter in the Drosophila eye discs [46]. In the future, it will be important to determine whether Ttk DNA binding sites are found in the Notch-responsive *stg *promoter as well. In addition, the binding sites of transcription factors that can interact with Ttk will be of interest, since Ttk can act as a DNA binding or non-binding repressor [47].

### JNK-pathway in cell cycle control

Previous work revealed that the JNK pathway is closely connected to cell cycle control [48]. For example, in fibroblasts the JNK pathway is critical for *cdc2 *expression and G2/M cell cycle progression [49]. In the case of the follicle cell mitotic-to-endocycle transition, we show that the JNK pathway is a critical positive controller of the mitotic cycles. Lack of JNK activity leads to a block in mitosis and initiation of premature endocycles. Conversely, lack of the negative regulator of the JNK-pathway, the phosphatase Puckered, results in a loss of endocycles. However, *puc *mutant clones do not consistently support extra divisions but might induce apoptosis as shown recently in disc clones [38].

**Figure 7:**
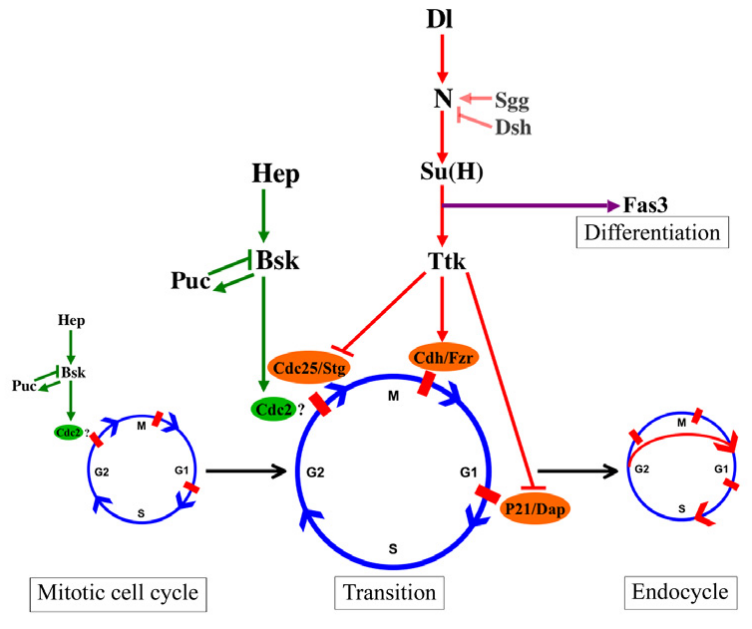
The JNK pathway promotes and maintains mitosis until the mitotic-to-endocycle transition point. At the transition, the Notch pathway acts through *ttk *on the cell cycle components *stg*, *dap*, and *fzr *in follicle cells to stop mitotic cycling and induce endocycling. Notch, which is modulated by Dl, dsh, and sgg, also plays a role in cell differentiation without the involvement of ttk, as shown by its effect on FAS3 expression.

These data are interesting in light of the results showing that the JNK pathway does not control the same cell cycle targets as the Notch pathway (Fig. 6), and could be explained by the following hypothesis (Fig. 7): the JNK-pathway positively regulates the mitotic cycles prior to stage 6 in follicle epithelial cells. This positive action on mitotic cycles is negatively short-circuited by the direct control of cell cycle regulators by the Notch pathway at stage 6 in oogenesis, resulting in the mitotic-to-endocycle transition (Fig. 7). Premature termination of the JNK pathway is sufficient to induce mitotic-to-endocycle transition (Fig. 6A–D). However, prolonged JNK activity, while disrupting endocycles, cannot maintain mitotic cycling efficiently, due to Notch action on *string*, *dacapo*, and *fzr*.

What then terminates JNK-pathway activity at stage 6 in oogenesis? Prolonged JNK activity (puc mutant clones) affects endocycles and the expression of pJNK and Puc subsides at stages 6–7 (Fig. 1H–I); results that both suggest the downregulation of JNK activity at the mitotic-to-endocycle transition. One possibility is that Notch activity downregulates the JNK pathway. However, at least Su(H)-dependent Notch activity does not regulate the JNK pathway, since no effect on *puckered *expression was observed in Su(H) mutant clones (data not shown). It is plausible that Su(H)-independent Notch activity regulates the JNK pathway in this context, as has been shown to be the case in dorsal closure [50]. Interestingly, Deltex might play a role in this Su(H)-independent Notch activity [51].

### Separation of differentiation and cell cycle control

An important question in analyzing the developmental control of cell cycle is whether the same signaling pathways control both differentiation and cell cycle, and if so, how the labor is divided. The Notch-dependent mitotic-to-endocycle transition is an example of such a question; Notch action in stage 6 follicle cells is critical for the cell cycle switch and for at least some aspects of differentiation. In this work we report the first component that separates Notch dependent cell cycle regulation from Fas3 marked differentiation; Ttk. In the *ttk *mutant clones, upregulation of FAS3, characteristic for Notch clones, is not observed. Therefore, Ttk constitutes a branch of Notch activity that might be solely required for cell cycle control in this context. However, we can not yet rule out Ttk's independent function. In the future, it will be important to understand whether signaling pathways in general show a clear separation of differentiation and cell cycle control on the level of downstream transcription factors.

Importantly, these and previous results have revealed the essential cell cycle regulators and their roles in controlling the Notch-dependent mitotic-to-endocycle switch [8,9,13,14]. The future challenge is to reveal the molecular connection between the Notch pathway, Ttk, and the critical cell cycle regulators.

## Methods

### Flies used

We used the following alleles in our studies: FRT 101 dsh^477^/FM7 [52](from Steve Cohen), FRT82B pygo^F66^, FRT82B pygo^F15 ^[53](from Xinhua Lin), FRT 82B pygo^S123^, y^1 ^sgg^M1-1 ^w^1 ^P{FRT(whs)}101/FM7a (Bloomington Stock Center, #5402), Bsk^170B ^FRT 40A/Cyo [54](from S. Noselli), FRT 82B ttk ^*1e11*^/TM6B [46], N^55e11 ^FRT101/FM7, y; Su(H)^047 ^FRT 40A/Cyo, dacapo^5gm.T:Hsap\MYC ^[55](from C. Lehner), pstgβ-E6.4 [56](string-lacZ from B. Edgar), w67c23 P{lacW}rapG0418/FM7c (fzr-lacZ, Bloomington Stock Center #12297), puc^A251^/TM3 Sb ser, w hep^r75^/FM7c [54](from S. Noselli), Mat-alpha4-Tub>Gal4-UP16/Cyo.

### Generation of follicle cell clones

We used the FLP/FRT system to generate follicle cell clones. Well-yeasted flies of the following genotypes were heat-shocked for 50–60 minutes at 37° two days in a row and allowed to develop on yeast for 2–5 days before dissection in PBS: sgg FRT101/Ubi-GFP FRT101; hs-FLP MKRS/+, dsh FRT101/Ubi-GFP FRT 101; hs-FLP MKRS/+, hs-FLP/+; FRT 82B pygo/FRT 82B Ubi-GFP, hs-FLP/+; ttk FRT 82B/Ubi-GFP FRT 82B, N FRT 101/Ubi-GFP FRT101; hs-FLP MKRS/+, hs FLP; FRT 82 puc [A251]/TM3 Sb ser, hs-FLP/+; Bsk^170B ^FRT 40/Ub-GFP FRT 40, hep^r75 ^FRT 101/Ubi-GFP FRT101; hs-FLP MKRS/+.

### Generation of transgenic pUASp-Delta flies and overproduction of Delta in the germ line

Full length Delta PCR products were synthesized using the forward primer GCTCTAGAAGCGACACTCAATC (Invitrogen) and the reverse primer GCTCTAGAGATGTCTCAATCGAT (Invitrogen) from the template EG194 (provided by Ed Giniger). PCR products were then digested with *XbaI *and cloned into the pUASp vector [57]. Results were confirmed via sequencing. The pUASp-Delta construct was injected into embryos, and two stable transformant lines were generated, pUASpDl-1 and pUASpDl-2. Mat-alpha4-Tub>Gal4-UP16/Cyo was used to drive expression of the pUASp-Dl constructs. Well-yeasted 1–5 day old flies were dissected and stained with Dl and PH3 antibodies.

### Antibody staining

For all antibodies, ovaries were dissected in phosphate-buffered saline (PBS) and fixed for 10 minutes in PBS containing 5% Formaldehyde. For all antibodies except pJNK, they were rinsed with PBT (PBS/0.2% Triton X-100) four times (15 minutes each) and blocked in PBTB (PBT, 0.2% BSA, 5% Normal Goat Serum) for one hour at room temperature. The tissue was incubated with primary antibodies overnight at 4°C. The next day they were rinsed with PBT four times (15 minutes each) and blocked in PBTB for one hour at room temperature. The ovaries were then incubated in secondary antibodies overnight at 4°C. The next day they were rinsed with PBT for 15 minutes, stained with DAPI (1 μg/ml in PBT) for 10 minutes, and rinsed with PBT two times (15 minutes each). Finally, ovaries were mounted onto slides in 70% glycerol, 2% NPG, 1× PBS. Samples stained with pJNK were processed in a similar manner, except that all washes and primary and secondary antibody incubations were done in BBT (PBT, 0.1% BSA, 250 mM NaCl). We used antibodies to the following proteins: Fasciclin3 (Developmental Studies Hybridoma Bank (DSHB), mouse, 1:20), CyclinB (DSHB, mouse, 1:20), phospho-histone3 (Upstate Biotechnology, rabbit 1:200), anti-β-gal (rabbit, 1:5000, Sigma), CyclinA (DSHB, mouse 1:15), cMyc (Calbiochem, mouse 1:100), Notch intra (DSHB, mouse 1:20), Ttk 69 and 88 [58](both rat 1:200, gifts from Paul Badenhorst), pJNK (Promega, rabbit 1:200), Delta (mouse, 1:3000). Images were collected on a 2-photon laser-scanning microscope (Leica TCS SP/MP).

### Statistical analysis

Statistical analysis for the data set was done using paired Student's t-test using Prism 4 for Macintosh (GraphPad software, San Diego, CA). Data comparisons were considered statistically significant if the p < 0.05.

## Supplementary Material

Additional File 1 Cut expression is down-regulated at mitotic-to-endocycle transition at stage 6 follicle cells.  (A, Aâ€™) Strong Cut-expression is observed in follicle cells prior to stage 6 and in polar cells after stage 6(Bâ€™). (B, Bâ€™) Cut down-regulation is normal in ttk-clones, except in the polar cell precursor group (Althauser et al., 2005; C, Câ€™). Click here for file
