# Supplementary figures and images for: Notch signaling through Tramtrack bypasses the mitosis promoting activity of the JNK pathway in the mitotic-to-endocycle transition of Drosophila follicle cells
Source: BMC Dev Biol. 2006 Mar 16;6:16. doi: 10.1186/1471-213X-6-16 (PMC1436016; doi:10.1186/1471-213X-6-16)

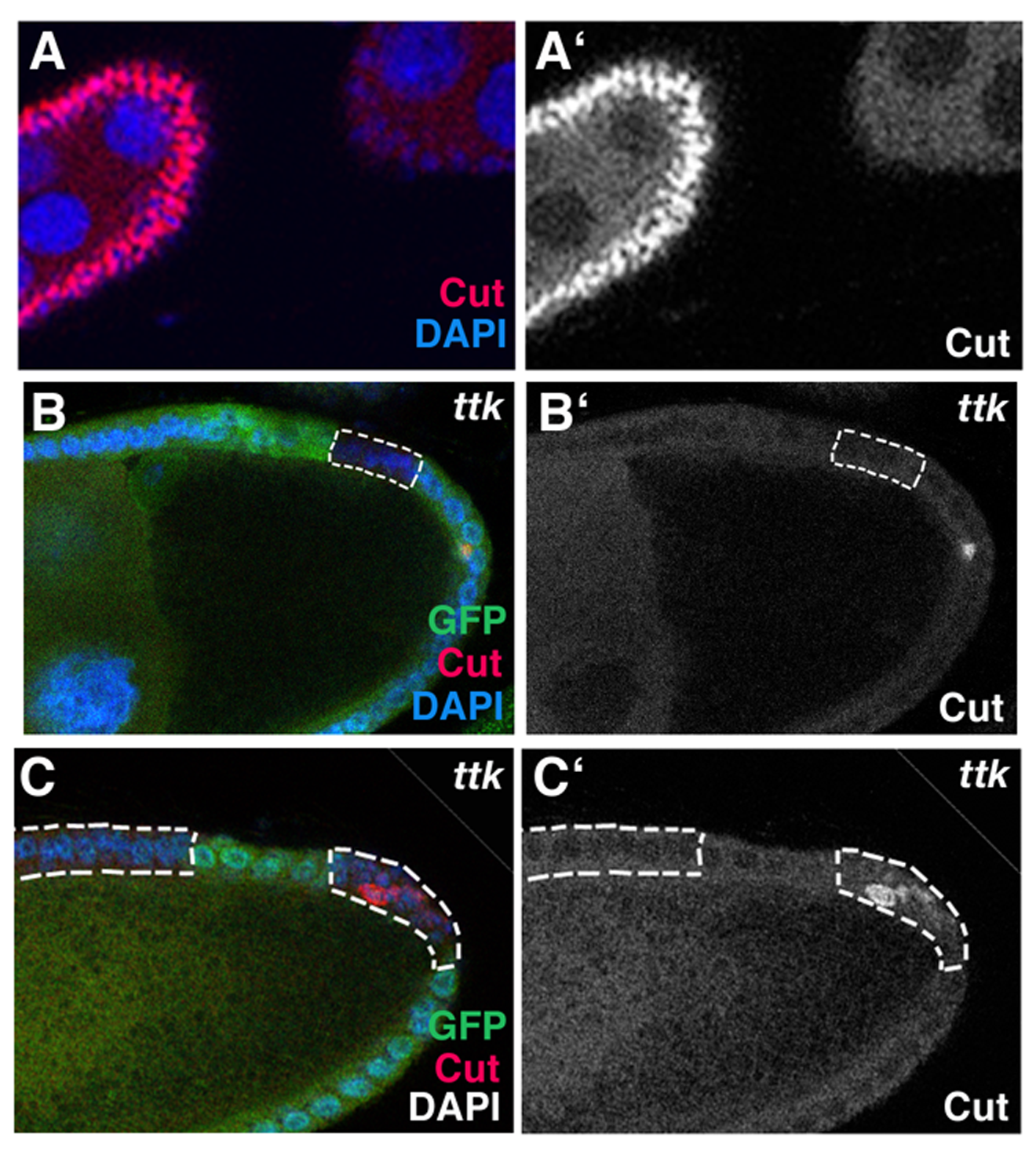

Supplement: Additional File 1 — Cut expression is down-regulated at mitotic-to-endocycle transition at stage 6 follicle cells. (A, Aâ€™) Strong Cut-expression is observed in follicle cells prior to stage 6 and in polar cells after stage 6(Bâ€™). (B, Bâ€™) Cut down-regulation is normal in ttk-clones, except in the polar cell precursor group (Althauser et al., 2005; C, Câ€™). [file 1471-213X-6-16-S1.tiff]
